# A Central Public Laboratory Clinic in Hospital and Private Practice

**Published:** 1913-12-13

**Authors:** David Walsh

**Affiliations:** Senior Physician, Western Skin Hospital, London, W.


### THE PLACE OF A CENTRAL PUBLIC LABORATORY CLINIC IN HOSPITAL AND PRIVATE PRACTICE.

By DAVID WALSH, M.D., Senior Physician, Western Skin Hospital, London, W.

By a "laboratory clinic" is meant an institution that combines the work of a clinical laboratory with the actual administration, when necessary, of technical methods of treatment, such as those by vaccines, intra-venous medication, and the like.

The rapid advances of medical science have necessitated the use of many complicated and costly methods of diagnosis. One has only to compare the practice of ten or fifteen years ago with regard, say, to enteric fever, tuberculosis, malaria, and venereal diseases, to realise how vital is the help of an expert pathologist in order to form an accurate diagnosis. Blood examinations are imperative in a large number of cases; sputum must be tested for micro-organisms; and a complicated series of investigations carried out with reference to many morbid processes.

The relation of general practice to advanced medical science is well illustrated in the special case of venereal disease, which, above all maladies, demands the services of the clinical laboratory. A great campaign is now on foot to rid the nation of that heavy burden, and a Royal Commission has been appointed to inquire into the subject. As medical men know, many persons thus affected come to the special hospitals for the skin, throat, eye, ear, and nervous system, and to those for women and children. Another large contingent applies to practitioners on the Insurance panel, and, generally, to all others who are called upon to treat poor patients. In the majority of cases the real nature of their malady is unsuspected by the patients themselves. Happily, it is now possible in obscure cases to ascertain by means of the Wassermann blood test and other methods of diagnosis whether syphilis is or is not actually present, and whether it has or has not been definitely driven out of the system. Moreover, the prospects of cure have been immensely quickened and aided by the introduction of Ehrlich's famous remedy known as "606," or "Salvarsan." Unfortunately, the cost of tests and of remedy is so great as to be almost prohibitive if used extensively in the small hospitals, and still more so among patients of the insured class. A similar observation applies to the bacteriological diagnosis of other maladies, and to the vaccine and other modern remedies needed for many infections of a non-venereal character.

### The Cost of Modern Methods.

The increased cost and complexity of modern scientific methods involve serious economic results. They threaten to undermine the whole fabric not only of the smaller voluntary charities, but also of the immense field covered by the National Insurance Act, and to a somewhat lesser extent by the Poor-Law Medical service. By denying the workman the chance of speedy relief from his illness it casts the burden of his support upon the public purse. In many ways it adds to the already heavy problem of how to deal with the waifs and strays of humanity. In the long run, however, the provision of modern scientific methods may be looked on as a sound investment, inasmuch as it lessens the ultimate burden upon the community of supporting its derelicts. The pressing problem of the moment is how to bring modern medical science to the homes of the poor.

As regards this question of advanced scientific method, existing hospitals may be divided sharply into two classes: first, those that possess well-appointed clinical laboratories, and, secondly, those that are unprovided in that particular way. The latter class are placed in this dilemma: either they must furnish their patients with medical service that is, scientifically speaking, behind the times, or they must send this special work at great cost to a private laboratory. Under the last-mentioned conditions it is obvious that physicians would naturally spare the resources of the hospital by limiting the number of investigations, even if no direct pressure were brought to bear upon them by the hospital management. Much the same may be said of the preparation of vaccines and of special methods of treatment.

### The Saving of a Central Laboratory.

Economically, as I have shown elsewhere,* there would be a great saving in the provision of a central laboratory available for a number of small medical charities. To that scheme I would now add the important suggestion that such a laboratory should be thrown open to private and to panel practice.

My scheme aims at the establishment of a central laboratory and clinic, intended primarily to supply the needs of the Metropolis, but capable of wider extension to the provinces later if such a course were desirable. To this institution all voluntary hospitals, panel and non-panel doctors would be entitled to send patients on certain simple conditions for the testing of blood and sputum: for bacteriological diagnosis: and, generally, for all kinds of clinical laboratory investigation. In addition the institute would, at the special request of the medical man concerned, provide and administer vaccines, tuberculin, salvarsan, and other highly specialised and often costly remedial agents.

An immediate result of the establishment of a central laboratory of the kind would be to bring under the best modern treatment a large amount of disease that is now being dealt with more or less ineffectively. Take the case of the panel doctor, into whose busy hands drift a large proportion of those patients whose maladies demand laboratory investigation. How, for instance, is he to determine

* "The Present Position of Dermatology."—*Medical Press and Circular*, January 29, 1913.

whether a doubtful rash is of tubercular, syphilitic, or other origin? Yet the decision may be vital to the health of the patient, not to mention the friends of the patient and the pockets of the ratepayers. What a boon it would be to the overworked panel doctor, harassed by many such perplexities in his day's work, were he able to send off his patients to have a diagnosis made or confirmed, to be followed, if necessary, by appropriate treatment! He himself has neither the leisure, nor the special experience, nor the means, nor the appliances for carrying out highly technical and costly modern scientific methods. In the case of the voluntary medical charities the assistance of a central laboratory could hardly be otherwise than valuable. Hospitals are often so crowded, especially in their out-patient departments, that it is impossible to give to individual patients the amount of time and care that is required if scientific justice is to be achieved in each case. In a word, to all medical men who have to see poor patients a central laboratory and clinic such as that under discussion should prove an invaluable aid to the proper performance of his duties.

### The Scheme in Outline.

What would be wanted to carry out such a scheme?

First of all, premises would have to be secured in a central situation convenient for the purpose of a laboratory and clinic, the prominent features of which would be:—

1. A well-equipped clinical laboratory with various departments, having a staff of expert pathologists at salaries ranging from, say, £150 to £250 per annum (one whole-time appointment at the lower figure to start with).

2. A clinical out-patient department for the administration of vaccines, salvarsan, and other special treatment by a staff of medical assistants, at salaries of £150 to £200 (part-time appointments).

3. Both the laboratory and the clinical departments would be placed under the charge of a medical superintendent at a salary of £300 to £400 per annum (part-time appointment).

4. The administrative staff would require a secretary, a matron, a nurse, a cook, wardmaid, and porter.

5. It would be well to have five to ten beds available for male and for female patients whom it might be advisable to keep under observation for one night after the administration of certain remedies by intravenous methods.

The cost of the premises and the equipment of the laboratory and of the organisation as a whole would not be great, as things go nowadays; certainly nothing like the vast sums that have been recently lavished upon more than one institution for special treatment, the therapeutic claims of which are still largely of a tentative character. On the other hand, the proposed laboratory and clinic would go straight to the heart of things and would from the first moment of its existence put into the hands of the medical profession the means of dealing with a vast amount of disease in its more insidious forms.

Sooner or later, it may be assumed with a fair amount of conviction, the State will be compelled to furnish some such free laboratory organisation if the principle underlying the National Insurance Act—namely, the supreme necessity of guarding the health of the individual citizen—is to be logically maintained.

For the funds necessary to the carrying out of the scheme I would apply to:

1. The philanthropic public.

2. The small hospitals, which would benefit largely from the laboratory clinic.

3. The King Edward and the other great distributing hospital agencies, inasmuch as the suggested laboratory clinic would (*a*) lessen the burden on the special and the smaller hospitals, and (*b*) greatly increase the efficiency of their work.

4. Subsidy from the State on the ground that the efficiency of the panel system would be greatly increased were a central laboratory and clinic available. (It is within the power of the Insurance Commissioners to sanction grants of the kind.)

5. The payment of small fees by institutions sending patients and by panel and non-panel patients on the guarantee of a medical man that the applicant is unable to pay ordinary medical fees, but is in a position to make a small contribution to the laboratory clinic. (Indigent persons would, of course, be treated free.)

It may be that amid the conflicting interests which mark the voluntary medical charities of the Metropolis a practical scheme such as that above outlined may not at present find a footing. On the other hand, it may perhaps win approval from one or more of those philanthropists whose generosity appears to be well nigh inexhaustible.

